# A transient DMSO treatment increases the differentiation potential of human pluripotent stem cells through the Rb family

**DOI:** 10.1371/journal.pone.0208110

**Published:** 2018-12-12

**Authors:** Jingling Li, Cyndhavi Narayanan, Jing Bian, Danielle Sambo, Thomas Brickler, Wancong Zhang, Sundari Chetty

**Affiliations:** 1 Department of Psychiatry and Behavioral Sciences, Stanford University School of Medicine, Stanford, California, United States of America; 2 Institute for Stem Cell Biology and Regenerative Medicine, Stanford University School of Medicine, Stanford, California, United States of America; University of Tampere, FINLAND

## Abstract

The propensity for differentiation varies substantially across human pluripotent stem cell (hPSC) lines, greatly restricting the use of hPSCs for cell replacement therapy or disease modeling. Here, we investigate the underlying mechanisms and demonstrate that activation of the retinoblastoma (Rb) pathway in a transient manner is important for differentiation. In prior work, we demonstrated that pre-treating hPSCs with dimethylsulfoxide (DMSO) before directed differentiation enhanced differentiation potential across all three germ layers. Here, we show that exposure to DMSO improves the efficiency of hPSC differentiation through Rb and by repressing downstream E2F-target genes. While transient inactivation of the Rb family members (including Rb, p107, and p130) suppresses DMSO’s capacity to enhance differentiation across all germ layers, transient expression of a constitutively active (non-phosphorylatable) form of Rb increases the differentiation efficiency similar to DMSO. Inhibition of downstream targets of Rb, such as E2F signaling, also promotes differentiation of hPSCs. More generally, we demonstrate that the duration of Rb activation plays an important role in regulating differentiation capacity.

## Introduction

Human pluripotent stem cells self-renew and differentiate into a variety of cell types, which make them an attractive source for cell replacement therapy and disease modeling [1]. However, an important issue that has challenged the stem cell field is the variability and bias in differentiation capacity observed across different hPSC lines, including both human embryonic and induced pluripotent stem cell lines [2–4]. Therefore, understanding the mechanisms regulating hPSC differentiation potential is of great value for regenerative medicine.

The retinoblastoma protein (Rb) plays a critical role in cell proliferation, differentiation, survival, and maintaining genomic stability [5–9]. During embryonic development when stem and progenitor cells divide and differentiate, Rb and its family members are present at varying levels and regulated in a transient manner [10,11]. This suggests that the timing and duration of Rb regulation may play an important role on stem cell differentiation. Here, we investigate the role of transient vs long term activation of Rb on hPSC maintenance and differentiation.

In a prior study, we showed that a simple 24h treatment of hPSCs with 1–2% DMSO prior to directed differentiation increases the differentiation potential of multiple cell lines across all germ layers [12]. This technique is now used by other laboratories in a variety of differentiation protocols and has been shown to increase hPSC differentiation towards numerous cell types, including hepatocytes, pancreatic progenitor cells, insulin-secreting β-cells, cardiomyocytes, neurons, contractile skeletal myotubes, brown adipose cells, intestinal epithelial cells, enterocytes, endothelial cells, and smooth muscle cells [13–23]. Treatment with DMSO is now also used in mouse and primate PSCs to increase multilineage differentiation capacity [15,18], suggesting that the treatment may be targeting a common mechanism across species. More recently, treatment of hPSCs with DMSO has also been shown to increase the efficiency of genome editing using Clustered Regularly Interspaced Short Palindromic Repeats (CRISPR)/CRISPR-associated protein-9 (Cas9) technology [24]. While the use of DMSO is now more widespread, the mechanism by which it regulates differentiation remain less understood and DMSO is well known to have pleiotropic effects on cells [25,26]. In conjunction with transiently manipulating Rb activity in hPSCs, we show that the DMSO treatment promotes differentiation through Rb. This study is the first to demonstrate that DMSO requires the activity of RB and its family members and downstream effectors in order to enhance differentiation. While knockdown of Rb and its family members can abolish DMSO’s ability to increase differentiation, activating Rb alone in a transient manner can mimic the DMSO treatment to promote differentiation. More generally, this study highlights the importance of transient rather than prolonged activation of Rb in promoting differentiation.

## Results

### Transient knockdown of Rb suppresses hPSC differentiation

**Fig 1:**
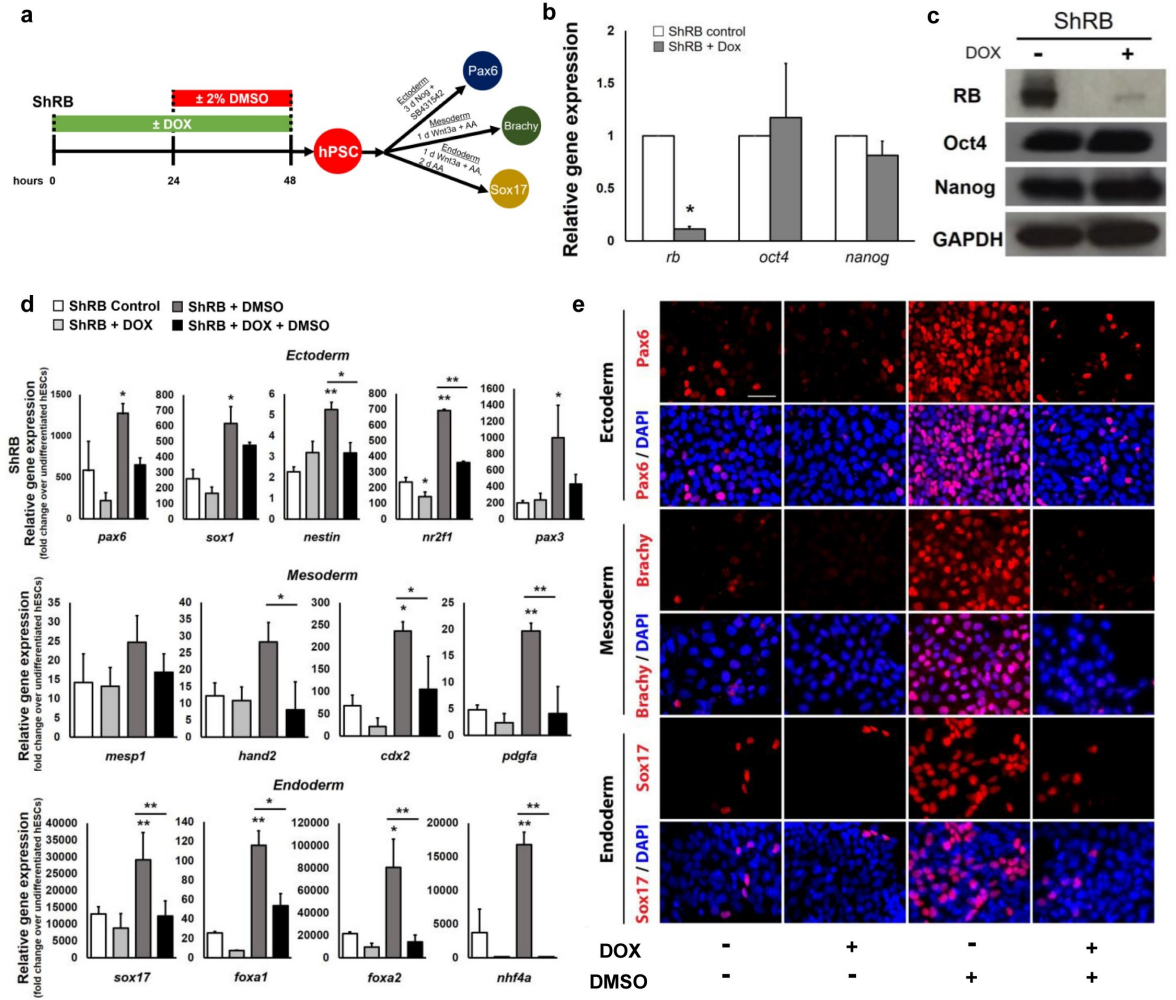
Knockdown of the Rb protein suppresses DMSO-induced improvements in hPSC differentiation. (a) Directed differentiation of the ShRB cell line into the three germ layers. (b) Quantitative real-time-PCR analyses of the expression levels of Rb and pluripotency genes in ShRB cells without DOX (ShRB control) and with DOX treatment (ShRB+Dox); * p ≤ 0.05 under two-tailed t-test. (c) Protein expression of RB and pluripotent markers, Oct4 and Nanog, by western blotting. GAPDH serves as a loading control. (d) Quantitative RT-PCR for lineage-specific genes and (e) immunostaining for *pax6* (ectoderm), *brachy* (mesoderm), and *sox1*7 (endoderm) following directed differentiation. Error bars, s.d. of 2–4 biological replicates. Scale bars, 100 μm. * p ≤ 0.05, ** p ≤ 0.01 under one-way ANOVA; Tukey’s test for multiple comparisons.

To investigate whether Rb function is necessary for hPSC differentiation, we used a doxycycline (DOX)-inducible hPSC line expressing short hairpin RNA against the Rb protein (ShRb) tagged with the green fluorescent protein (GFP) to knock down the Rb protein in hPSCs [27]. Following DOX treatment of hPSCs for 48 hours (Fig 1A), ShRB cells displayed a significant decrease in RB expression at both the mRNA and protein levels relative to cells without DOX treatment (Fig 1B and 1C). Oct4 and Nanog were found to be expressed at both the mRNA (Fig 1B) and protein (Fig 1C) levels as in control cells with colony morphology also resembling hPSCs (S1A Fig). High levels of Oct4, SSEA4, and TRA-1-60 were also detected by immunostaining of ShRB hPSCs (S1C–S1E Fig), and GFP expression provided additional confirmation of Rb knockdown in DOX-treated ShRB cells (S1B Fig). Together, these results indicate that ShRB cells retain pluripotency characteristics following transient inactivation of Rb.

Though a 24h treatment of hPSCs with 2% DMSO activates Rb and increases the multilineage differentiation capacity of hPSCs [12], it remains unknown whether Rb is required for these DMSO mediated effects. We transiently knocked down Rb by treating with DOX for 48h and assessed the differentiation capacity of ShRB cells treated with and without 2% DMSO for 24h prior to directed differentiation into the three germ layers (Fig 1A) following established protocols [12,28]. The mRNA (Fig 1D) and protein levels (Fig 1E) of several germ layer specific genes [29] were assessed by qRT-PCR and immunostaining. Treatment with DMSO significantly increased differentiation across all germ layers relative to untreated controls. However, transient knockdown of Rb in ShRB DOX-treated cells significantly reduced the capacity of the DMSO treatment to improve differentiation (Fig 1D and 1E, S2A and S3A Figs). For some germ layers, a moderate degree of differentiation persists in ShRB DOX-treated cells, potentially indicating that knockdown of Rb alone may not be sufficient to completely abolish differentiation. Nonetheless, across all germ layers, transient knockdown of Rb significantly reduced the effect of DMSO on the differentiation potential of hPSCs. Consistent with a prior study [27], knockdown of Rb alone did not alter the distribution of hPSCs in the cell cycle. The enrichment of cells in the G1 phase following DMSO treatment was moderately reduced but remained high in DMSO- and DOX-treated ShRB cells (S4A Fig).

### Transient knockdown of all three Rb family members regulates hPSC differentiation

**Fig 2:**
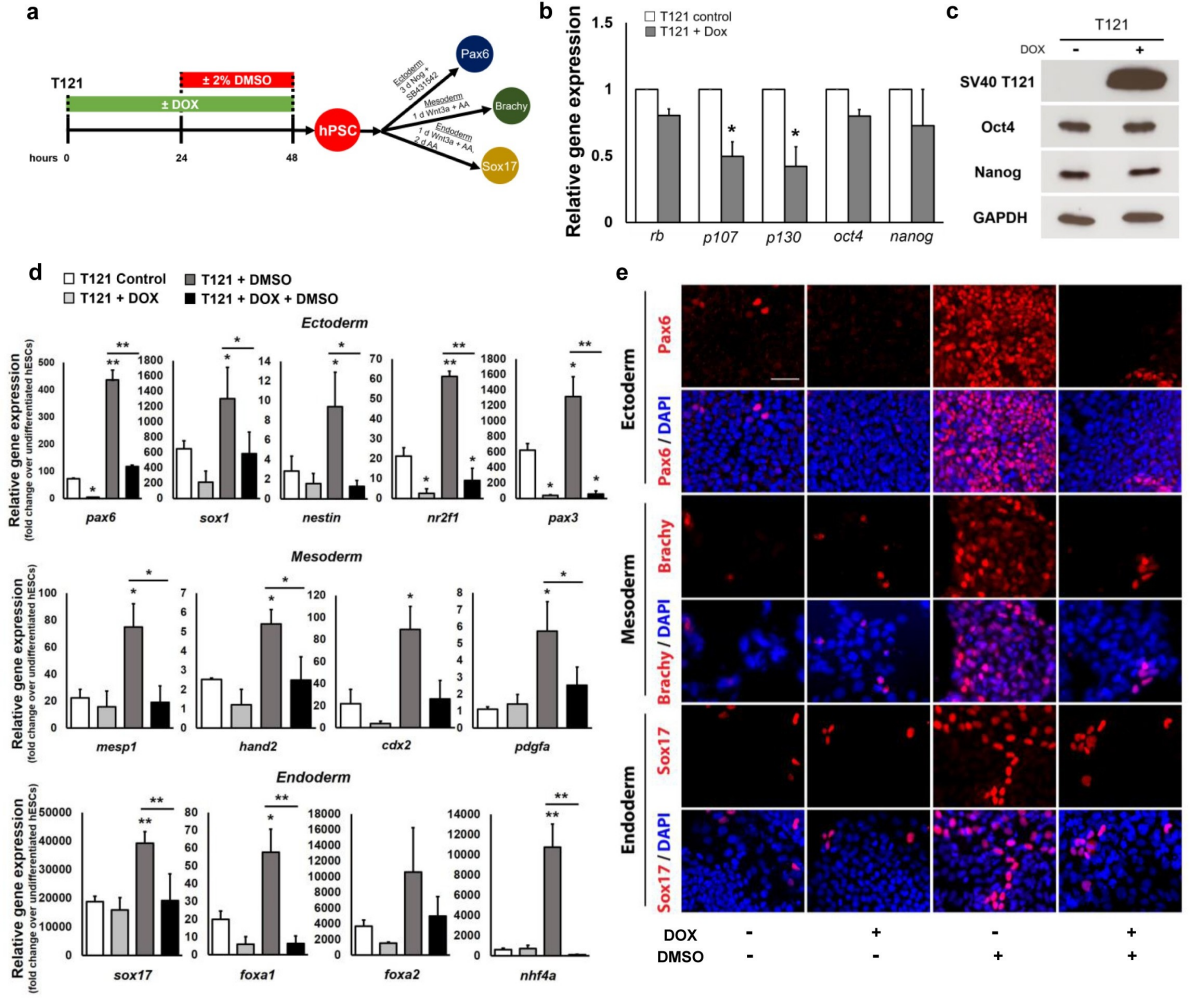
Inactivation of the Rb family attenuates DMSO-induced improvements in hPSC differentiation. (a) Directed differentiation of the T121-hPSC line, an inducible cell line that inactivates all three Rb family members, into the three germ layers. (b) Quantitative RT-PCR analyses of the expression levels of Rb, p107, p130, and pluripotency genes in T121 cells without DOX (T121 control) and with DOX treatment (T121+Dox); * p ≤ 0.05 under two-tailed t-test. (c) Protein expression of the SV40 T antigen (T121) and pluripotent markers, Oct4 and Nanog, by western blotting. GAPDH serves as a loading control. (d) Quantitative RT-PCR for lineage-specific genes and (e) immunostaining for *pax6* (ectoderm), *brachy* (mesoderm), and *sox17* (endoderm) following directed differentiation. Error bars, s.d. of 2–4 biological replicates. Scale bars, 100 μm. * p ≤ 0.05, ** p ≤ 0.01 under one-way ANOVA; Tukey’s test for multiple comparisons.

Loss of Rb expression may stimulate a compensatory response by other Rb family members. To investigate this further, we next used an inducible hPSC line infected with lentiviral vectors expressing the dl1137 mutant of SV40 T antigen (T121) that binds and functionally inactivates all members of the Rb family in hPSCs [27]. Treatment with DOX (Fig 2A) resulted in a downregulation of Rb, p107, and p130 expression at the mRNA levels (Fig 2B) and showed clear protein expression of T121 by western blot (Fig 2C), suggesting successful inactivation of all three Rb family members. Oct4 and Nanog, were not significantly regulated at the mRNA (Fig 2B) and protein (Fig 2C) levels by qRT-PCR and western blot, respectively, following SV-40 induction, suggesting that transient inactivation of the Rb family members does not alter the expression of these pluripotency factors. While the DMSO treatment enriches hPSCs in the G1 phase of the cell cycle, inactivation of the Rb family reduced the enrichment of cells in G1 in DMSO-treated T121-expressing cells (S4B Fig), suggesting that T121-expressing hPSCs may be insensitive to G1 arrest signals. Nonetheless, transient treatment with DOX and/or DMSO of T121 hPSCs did not significantly alter proliferative capacity, cell growth, and cell death as total cell number and Ki67 expression remained consistent across conditions (S5A–S5D Fig).

To test whether inactivation of all three Rb family members affects the enhancement of differentiation capacity by DMSO, we directly differentiated control and T121-expressing cells with or without a 24 hour 2% DMSO pre-treatment towards all three germ layers (Fig 2A). Treatment with DMSO significantly increased expression of several germ layer specific genes at the mRNA and protein levels (Fig 2B and 2E, S2B and S3B Figs). However, transient inactivation of the Rb family significantly attenuated the capacity of DMSO-treated T121-expressing cells to differentiate towards all germ layers. Together, these results demonstrate an important role for Rb, p107, and p130 function in promoting hPSC differentiation.

### Transient activation of Rb promotes hPSC differentiation

**Fig 3:**
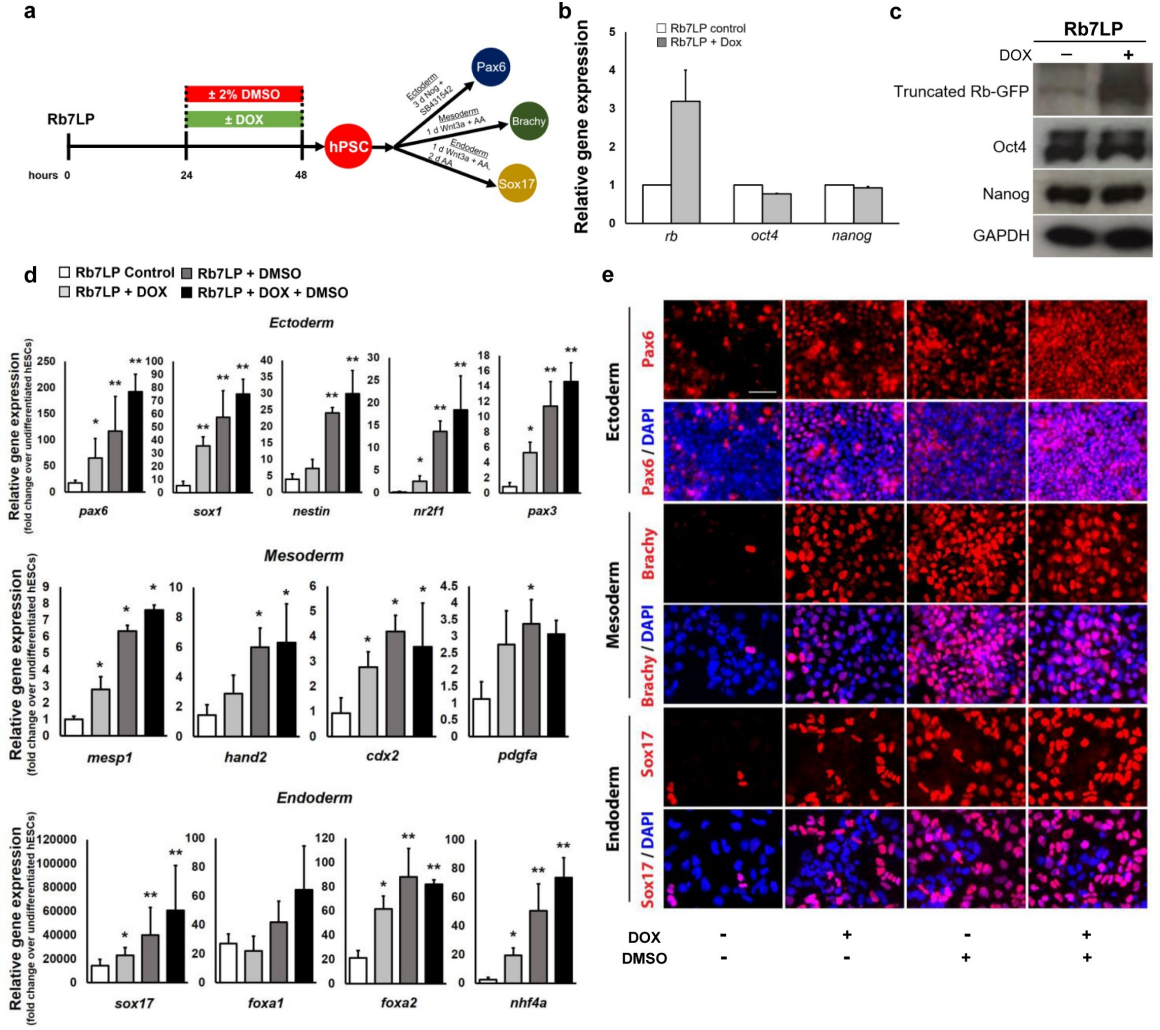
Activation of the Rb protein mimics the DMSO effects and increases the differentiation capacity of hPSCs. (a) Directed differentiation into the three germ layers of the dox-inducible Rb7LP cell line, which expresses the active non-phosphorylatable form of Rb, and compared with control and 2% DMSO-treated cells. (b) Quantitative RT-PCR analyses of the expression levels of Rb and pluripotency genes in Rb7LP cells without DOX (Rb7LP control) and with a 24h DOX treatment (Rb7LP+Dox). (c) Protein expression of the truncated Rb-GFP protein and pluripotent markers, Oct4 and Nanog, by western blotting. GAPDH serves as a loading control. (d) Quantitative RT-PCR for lineage-specific genes and (e) immunostaining for *pax6* (ectoderm), *brachy* (mesoderm), and *sox17* (endoderm) following directed differentiation. Error bars, s.d. of 4–6 biological replicates. Scale bars, 100 μm. * p ≤ 0.05, ** p ≤ 0.01 under one-way ANOVA; Tukey’s test for multiple comparisons.

Thus far, the data show that knockdown of Rb and its family members abrogates the improvements in hPSC differentiation efficiency induced by a 24h DMSO pre-treatment. We next investigated whether transiently activating Rb would mimic the DMSO effects and increase hPSC differentiation potential. To transiently activate Rb, we used a DOX-inducible cell line which expresses a constitutively active, non-phosphorylatable form of Rb tagged with the GFP protein (Rb7LP-GFP) [27] (Fig 3A). Following a 24h treatment with DOX, the expression of Rb significantly increased in Rb7LP cells compared to control cells (Fig 3B), as well as levels of the truncated Rb-GFP protein (activated Rb) (Fig 3C). Equivalent expression levels of Oct4 and Nanog were observed in control and Rb7LP-induced cells (Fig 3B and 3C), indicating that transient activation of Rb does not significantly alter the pluripotent state of hPSCs. Similar to the DMSO treatment, activation of Rb following DOX treatment also increased the proportion of hPSCs in the G1 phase of the cell cycle (S4C Fig) and showed a reduction in the phosphorylation status of Rb (S4D Fig). Transient treatment with DOX and/or DMSO of Rb7LP hPSCs also did not significantly alter proliferative capacity, cell growth, and cell death as total cell number and Ki67 expression remained consistent across conditions (S5E–S5H Fig).

We next investigated whether transiently overexpressing the non-phosphorylatable active Rb would increase the differentiation potential of hPSCs. We treated the Rb7LP cells with DOX and/or 2% DMSO for 24 hours prior to directed differentiation into each of the three germ layers (Fig 3A). Across all germ layers, both the expression and number of differentiated cells were significantly increased following activation of Rb relative to control cells (Fig 3D and 3E, S2C and S3C Figs). Fold changes in mRNA expression levels for several lineage-specific markers and genes were significantly upregulated in DOX-treated Rb7LP cells relative to control cells and reached levels near DMSO-treated hPSCs. Hence, activating Rb by promoting its de-phosphorylation mimics the DMSO effects by enriching cells in G1 and increasing subsequent differentiation potential. Treatment with both DOX and DMSO for 24h also improved differentiation potential relative to control cells (Fig 3D and 3E, S2C and S3C Figs), but expression levels of many germ layer specific genes were comparable to DMSO treatment alone and did not induce further improvements. Together, these results parallel those that occur during normal development in which the amount of hypophosphorylated, active RB increases at the onset of differentiation [10].

Activating Rb throughout the differentiation protocol with continued DOX treatment reduced differentiation relative to transient activation, as shown for the ectodermal lineage (S6 Fig). Following directed differentiation, we assessed the mRNA expression of two neuroectodermal genes, Sox1 and Pax6 (S6A Fig). Both genes showed reduced expression relative to DMSO-treatment and a transient DOX treatment (S6B Fig), suggesting an important role for transient activation of Rb in increasing the differentiation capacity of hPSCs.

### Inhibition of the E2F pathway promotes hPSC differentiation

**Fig 4:**
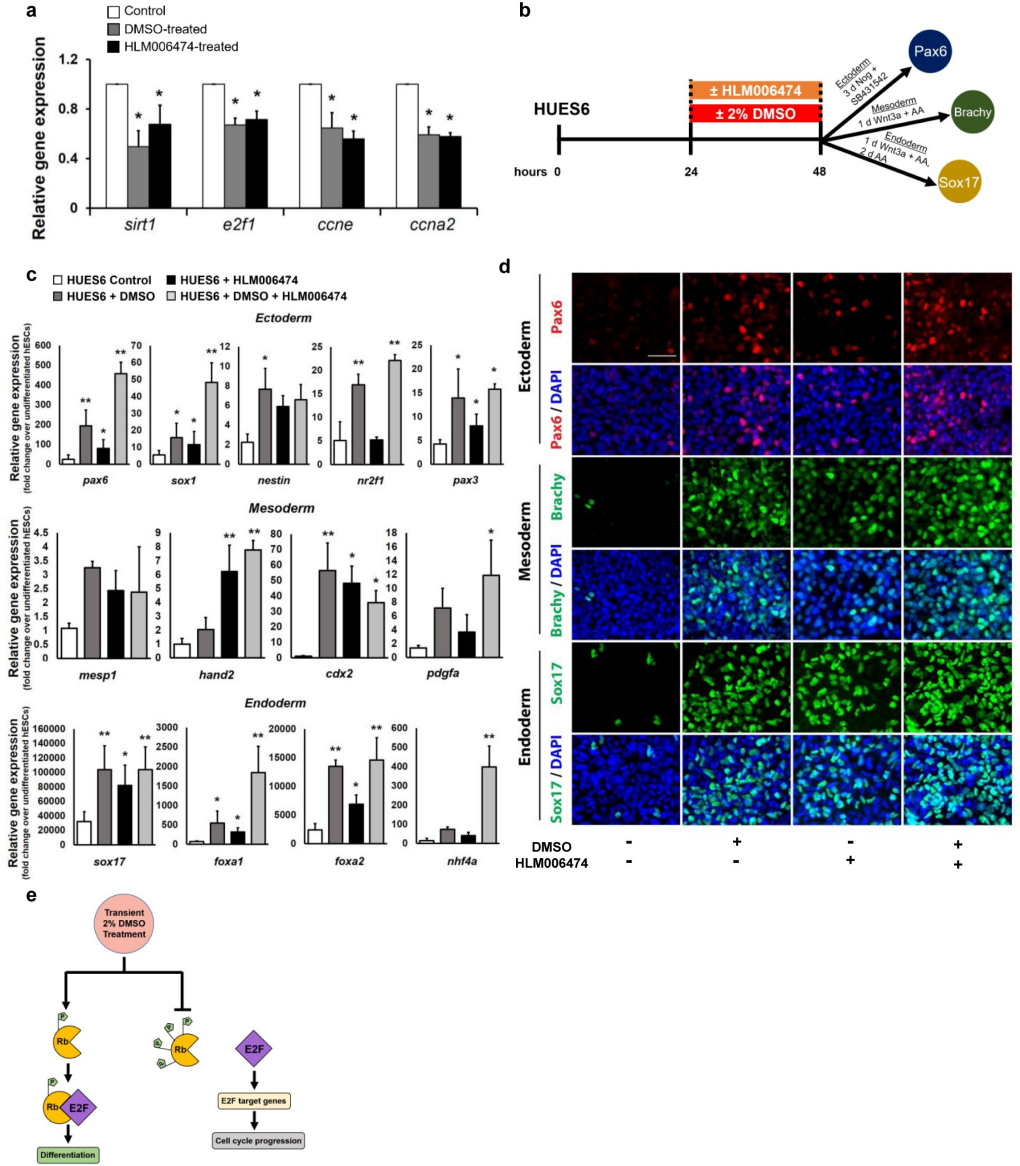
Inhibition of the E2F pathway increases the differentiation capacity of hPSCs. (a) The hPSC line HUES6 treated with 2% DMSO or 30 μM of the E2F inhibitor HLM006474 for 24 hours. Quantitative RT-PCR results of expression levels of E2F-target genes relative to untreated control cells. (b) Directed differentiation into the three germ layers of HUES6 cells pre-treated with 2% DMSO and/or 30μM E2F inhibitor HLM006474 for 24 hours. (c) Quantitative RT-PCR for lineage-specific genes and (e) immunostaining for *pax6* (ectoderm), *brachy* (mesoderm), and *sox17* (endoderm) following directed differentiation. (e) Schematic model showing that the transient DMSO treatment promotes hypophosphorylation of Rb and subsequent binding to E2F to increase differentiation potential (left) as opposed to increasing hyperphosphorylation of Rb and activating E2F and its downstream target genes to promote cell cycle progression (right). Error bars, s.d. of 4–6 biological replicates. Scale bars, 100 μm. * p ≤ 0.05, ** p ≤ 0.01 under one-way ANOVA; Tukey’s test for multiple comparisons.

The active hypophosphorylated Rb physically binds to the E2F transactivation domain to arrest cells in the G1 phase and prevent progression towards S phase [30–34]. As a result, activation of Rb decreases activity of the E2F pathway by suppressing E2F target genes [27,32,35–37]. If the DMSO treatment functions through the Rb-E2F pathway, we hypothesized that the DMSO treatment would downregulate E2F-associated genes and that inhibition of the E2F pathway would similarly enhance the differentiation capacity of hPSCs. We first assessed whether a 24h DMSO-treatment regulates the expression of E2F target genes. As a comparison, we also directly inhibited the E2F pathway using a small molecule E2F inhibitor HLM006474 [27,38,39] (30 μM) for 24 hours in the HUES6 hESC line, a cell line with a low propensity for differentiation[12,40]. In both DMSO-treated and HLM006474-treated HUES6 cells, the expression of E2F target genes [41,42], including *sirt1*, *e2f1*, *ccne*, and *ccna*, were significantly downregulated (Fig 4A). We next treated HUES6 cells with 2% DMSO and/or 30 μM HLM006474 for 24 hours and then directly differentiated the cells into the three germ layers to assess differentiation potential (Fig 4B). Across all germ layers, the DMSO treatment significantly improved the differentiation capacity (Fig 4C and 4D, S2D and S3D Figs). Similarly, repressing E2F activity through treatment with HLM006474 significantly increased differentiation across all germ layers promoting enhanced expression of several lineage-specific markers (Fig 4C and 4D, S2D and S3D Figs). For some genes, combining treatment with DMSO and HLM006474 increased expression levels further (Fig 4C and 4D, S2D and S3D Figs), potentially indicating a beneficial effect on cell lines with very low propensity. When E2F inhibition was extended by treating with HLM006474 prior to and during the differentiation protocol, the capacity for differentiation was suppressed compared to a transient treatment (S7 Fig). Similar to long term activation of Rb in the Rb7LP line, both Sox1 and Pax6 showed reduced expression levels relative to DMSO-treatment and a transient treatment with HLM006474 following differentiation into the ectodermal germ layer (S7A and S7B Fig). Together, these results provide further support that the DMSO treatment acts through the Rb-E2F pathway to regulate PSC differentiation (Fig 4E). More generally, these results highlight that *transient*, and not prolonged, activation of Rb and inhibition of downstream targets is important in increasing the differentiation capacity of hPSCs.

## Discussion

Differentiating hPSCs into desired lineages has tremendous value for cell replacement therapy and disease modeling. The Rb protein plays an important role in various cellular processes, including cell division, differentiation, senescence, apoptosis, and DNA damage and repair [7,10,43,44]. Using many different techniques to manipulate Rb and its activity in hPSCs, we demonstrate that a short transient regulation of Rb in hPSCs for 24 hours has a significant impact on their capacity to differentiate towards all germ layers. This study provides evidence that treatment with DMSO enhances differentiation through the Rb-E2F pathway. Prior work has shown that the phase of the cell cycle, particularly G1, plays an important role in enhancing differentiation [39,45–52]. However, Rb remains in a phosphorylated state throughout the cell cycle of PSCs and therefore this shortens the G1 phase and limits the window of opportunity for differentiation [53–57]. Here, we identify chemical and genetic tools for transiently activating Rb to regulate differentiation.

Regulating Rb using these agents or other means at subsequent stages of directed differentiation or to suppress tumorigenicity could also have important applications for regenerative medicine. More importantly, the effects reported here parallel those that occur during normal embryonic development where a transient surge in activated Rb coincides with differentiation and lineage specification [10,11]. It would be interesting to investigate whether similar mechanisms related to Rb activity regulate CRISPR/Cas9 genome editing in response to DMSO treatment [24].

## Materials and methods

### Cell maintenance

All cell lines were cultured and maintained at standard conditions at 37 ^o^C, 5% CO_2_. hPSCs were maintained and expanded in mTeSR medium (Stem Cell) on Matrigel-coated plates. Fresh mTeSR medium was replaced every 24 hours. To passage cell lines, TrypLE (Invitrogen) was applied to detach cells.

The cell lines analyzed in this study were the ShRB, T121, and Rb7LP inducible cell lines that were previously generated in the H9 hESC line (Wicell) [27] and the HUES6 hESC line[12,40]. Prior to pluripotent analyses or differentiation, cells were expanded on maintenance Matrigel (Corning) with 10 μM Y27632 (Rock Inhibitor; Stemgent) and treated with one of the following conditions:

#### Doxycycline treatment

ShRB, T121 and Rb7LP hESCs were plated at a density of 1 million per well of a Matrigel-coated six-well plate in mTeSR medium with 10 μM Y27632. Doxycycline (1 μg/ml) was added to ShRB and T121 cells on the day of plating. Fresh mTeSR containing doxycycline was replaced after 24 hours. For the Rb7LP cells, 1 μg/ml doxycycline was applied the day after plating. Cells were either harvested for pluripotent analyses or directly differentiated into the three germ layers after doxycycline treatment.

#### DMSO treatment

Cells were treated with 2% DMSO in mTeSR medium for 24 hours before the onset of differentiation.

#### E2F inhibitor treatment

Cells were treated with 30μM HLM006474 (EMD Millipore), an E2F inhibitor, in mTeSR medium for 24 hours before the onset of differentiation or throughout differentiation.

### Differentiation assays

To induce differentiation into the ectodermal, mesodermal and endodermal germ layers, cells were directly differentiated as follows:

#### Ectoderm

Cells were cultured in Knockout-DMEM (Invitrogen) containing 10% Knockout Serum Replacement (Invitrogen), Noggin (500 ng/ml; R&D Systems), SB431542 (10 μM; Tocris) for 3 days. The medium was removed and replaced with fresh medium every 24 hours.

#### Endoderm

Cells were cultured in RPMI medium (Invitrogen), supplemented with Wnt3a (20 ng/ml; R&D Systems) and Activin A (100 ng/ml; R&D Systems) for 24 hours and subsequently in RPMI medium containing Activin A (100 ng/ml) for 2 days.

#### Mesoderm

Cells were cultured in advanced RPMI medium (Invitrogen) supplemented with Wnt3a (20 ng/ml; R&D Systems) and Activin A (100 ng/ml; R&D Systems) for 1 day.

### RNA isolation and quantitative real-time PCR

Cell pellets were collected and the total RNA were isolated using RNeasy Mini Kit (QIAGEN) according to the protocol provided by the manufacturer. After determining the concentration and purity of RNA by the NanoDrop spectrophotometer, reverse transcription was conducted using SuperScript IV VILO Master Mix with ezDNase (Thermo Fisher) to synthesize cDNA.

Quantitative RT-PCR was performed using the SYBR green system. One reaction included SYBR green mix (Applied Biosystems), the forward and reverse target gene primers, and 100–120 ng cDNA. The ABI 7500 Real-Time PCR machine was used to run the qRT-PCR experiment. The level of RNA transcripts was analyzed using the ΔΔCT method. Gene expression was subsequently normalized based on the housekeeping GAPDH expression. The primers used in this study are listed in S1 Table.

#### Immunoblotting

Cells were lysed in the RIPA lysis Buffer (Thermo Scientific) supplemented with 1X Phosphatase Inhibitor (Thermo Scientific) and Protease Inhibitor (Thermo Scientific). The protein lysates were collected following the centrifugation.

The proteins were mixed with Novex Tris-Glycine SDS sample buffer (Invitrogen) and separated on the 8% Tris-glycine gels (Invitrogen). The iBlot Gel Transfer system (Invitrogen) was used to transfer protein onto the nitrocellulose membrane (Invitrogen). Subsequently, the membranes were blocked in 5% nonfat dry milk in Tris-buffered saline (TBS)/0.1% Tween-20 (Cell signaling Technology) for one hour. Primary antibodies were then diluted in the blocking solution and applied overnight at 4 ^o^C. The antibodies used in this study were: GAPDH (the loading control; 1:1000; Millipore); Oct3/4 (1:200; Santa Cruz); Nanog (1:1000; Cell signaling Technology); Rb (1:1000; Cell signaling Technology); SV-40 T121 (1:1000; Abcam); and GFP (1:200; Santa Cruz). After the membranes were washed in TBS/0.1% Tween-20 at room temperature on the second day, HRP-conjugated secondary antibodies were diluted in blocking solution (1:1000, Cell Signaling) and added to the membranes for one hour. ECL-Prime Western Blotting Detection Reagent (GE Healthcare) was used for signal detection.

#### Immunocytochemistry

Cells were fixed in 4% paraformaldehyde (Electron Microscopy Sciences) for 20 minutes at room temperature. Subsequently, the cells were rinsed in PBS and blocked in 5% normal donkey serum (Jackson ImmunoResearch) /0.3% TrionX-100 in PBS for one hour. Primary antibodies were prepared at a 1:500 dilution in the block solution and applied to cells overnight at 4°C. All primary antibodies used in this study were: GFP (Santa Cruz); Ki67 (Abcam); Oct3/4 (Santa Cruz); SSEA4 (Invitrogen); TRA-1-60 (Invitrogen); Brachy (R&D Systems); Pax6 (Santa Cruz); Sox1 (R&D Systems); and Sox17 (R&D Systems). After overnight incubation in primary antibodies, the cells were washed in PBS/0.3% TrionX-100 and incubated in fluorescently tagged secondary antibodies, Alexa-Fluor goat/donkey-anti-primary antibody species IgG 488 or 594 (Life Technologies), for 1h at room temperature and diluted at 1:500 in PBS/0.3% TrionX-100. DAPI (4,6-diamidino-2-phenylindol, Life Technologies) was used as a nuclear dye to stain all cells.

#### Image acquisition and quantification

Following directed differentiation into the three germ layers, 10x fields per well were acquired and quantified. Cells labeled by Pax6, Brachyury, or Sox17 antibody staining and total cell number (based on DAPI nuclei staining) were quantified using automated software in ImageJ to obtain percentages of target cell types.

#### Assessment of cell death and growth

To assess the degree of cell death, cells were harvested following treatment with and without DOX and resuspended in 1 ml PBS. 10 μl of Trypan Blue (Invitrogen) was mixed with 10 μl of cells. Subsequently, 10 μl of the mixed solution was loaded onto the Countess cell counting chamber slide (Invitrogen) and inserted into the Countess II FL Automated Cell Counter (Invitrogen). The percentage of dead or nonviable cells was quantified by the automated system using the trypan blue exclusion assay. To assess cell growth, the initial cell plating density was recorded for each cell line using the Countess II FL Automated Cell Counter. Following the Doxycycline (DOX) treatment, control and DOX-treated cells were collected and recorded for total cell number using the Countess II FL Automated Cell Counter. The percentage increase in cell number was calculated as: ((final cell number–initial cell number at plating)/initial cell number at plating) * 100%.

#### Cell cycle analysis

The cells were collected and fixed in 70% ethanol for 2 hours at 4°C. Cells were then centrifuged and pelleted and subsequently washed twice with PBS. The cells were resuspended in DAPI/PBS/0.1% TritonX-100 staining solution for 30 min. Stained samples were run on a flow cytometer adjusted for UV excitation to measure DAPI fluorescence at blue wavelengths.

#### Statistical analysis

For all the variables in this study, means and standard deviations were calculated. A one-way ANOVA followed by a Tukey’s *post hoc* test was used to determine statistical significance. For comparisons between two groups, the unpaired two-tailed Student’s *t*-test was used to determine statistical significance. P value ≤ 0.05 was considered statistically significant.

#### Regulatory and institutional review

All human pluripotent stem cell experiments were conducted in accord with experimental protocols approved by the Stanford Stem Cell Research Oversight (SCRO) committee.

## Supporting information

S1 Fig Knockdown of the Rb protein in hPSCs. Representative images of (a) phase-contrast hPSC colonies and (b) GFP expression following DOX induction of the ShRB cell line. Immunostaining for (c) Oct4, (d) SSEA-4, and (e) TRA-1-60 in control (-DOX) and ShRB (+DOX) cells. Scale bars, 100 μm.
(TIF)

S2 Fig Regulation of the Rb pathway alters directed differentiation potential of hPSCs. Percentage of hPSCs differentiating into Pax6+ ectodermal, Brachyury (Brachy)+ mesodermal, or Sox17+ endodermal cells following directed differentiation into each germ layer in the (a) ShRB, (b) T121, and (c) Rb7LP cell lines with and without DOX treatment and a 24h 2% DMSO treatment and the (d) HUES6 cell line pre-treated with and without 30μM E2F inhibitor HLM006474 and a 24h 2% DMSO treatment. Error bars, s.d. of 3–6 biological replicates. * p ≤ 0.05, ** p ≤ 0.01 under one-way ANOVA; Tukey’s test for multiple comparisons.
(TIF)

S3 Fig Transient activation of Rb or E2F inhibition increases expression of ectodermal genes. Immunostaining for Sox1 following directed differentiation of the (a) ShRb, (b) T121, and (c) Rb7LP cell lines into the ectodermal germ layer. (d) Immunostaining for Sox1 following directed differentiation into the ectodermal germ layer of the HUES6 cell line pre-treated with and without 30μM E2F inhibitor HLM006474 and a 24h 2% DMSO treatment.
(TIF)

S4 Fig Regulation of Rb alters the distribution of hPSCs in the cell cycle. Distribution of hPSCs in the G1, S, and G2/M phases of the cell cycle in the (a) ShRB, (b) T121, and (c) Rb7LP cell lines with and without DOX treatment and a 24h 2% DMSO treatment. (d) Western blot showing the levels of hyperphosphorylated Rb in Rb7LP cells with and without DOX treatment compared to DMSO-treated cells. ppRB, hyperphosphorylated Rb. GAPDH serves as a loading control.
(TIF)

S5 Fig Transient regulation of Rb does not alter proliferative capacity or viability of hPSCs. (a) Immunostaining for the proliferation marker Ki67 in T121 cells with and without DOX treatment and a 24h 2% DMSO treatment. (b) Percentage of dead cells of T121 cells following treatment with and without DOX and a 24h 2% DMSO treatment using the trypan blue exclusion assay. (c) Total cell numbers of T121 cells following treatment with and without DOX and a 24h 2% DMSO treatment. (d) Percentage increase in total cell number following treatment with and without DOX and a 24h 2% DMSO treatment relative to initial plating density in the T121 cell line. (e) Immunostaining for Ki67 in Rb7LP cells with and without DOX treatment and a 24h 2% DMSO treatment. (f) Percentage of dead cells of Rb7LP cells following treatment with and without DOX and a 24h 2% DMSO treatment using the trypan blue exclusion assay. (g) Total cell numbers of Rb7LP cells following treatment with and without DOX and a 24h 2% DMSO treatment. (h) Percentage increase in total cell number following treatment with and without DOX and a 24h 2% DMSO treatment relative to initial plating density in the Rb7LP cell line. Error bars, s.d. of 3–6 biological replicates.
(TIF)

S6 Fig Transient activation of Rb increases the differentiation capacity of hPSCs. (a) Directed differentiation into the ectodermal germ layer of the dox-inducible Rb7LP cell line, which expresses the active non-phosphorylatable form of Rb, and compared with control and 2% DMSO-treated cells. Treatment with DOX was for 24h prior to directed differentiation (Transient DOX-treated) or for 24h prior to directed differentiation and throughout the ectodermal differentiation (Long-term DOX-treated). (b) Quantitative RT-PCR analyses of sox1 and *pax6* expression following differentiation into the ectodermal germ layer. Error bars, s.d. of 3–5 biological replicates. * p ≤ 0.05, ** p ≤ 0.01 under one-way ANOVA; Tukey’s test for multiple comparisons.
(TIF)

S7 Fig Transient E2F inhibition increases the differentiation capacity of hPSCs. (a) Directed differentiation into the ectodermal germ layer of HUES6 cells treated with HLM006474 compared with control and 2% DMSO-treated cells. Treatment with HLM006474 was for 24h prior to directed differentiation (Transient HLM006474-treated) or for 24h prior to directed differentiation and throughout the ectodermal differentiation (Long-term HLM006474-treated). (b) Quantitative RT-PCR analyses of sox1 and *pax6* expression following differentiation into the ectodermal germ layer. Error bars, s.d. of 2–5 biological replicates. * p ≤ 0.05, ** p ≤ 0.01 under one-way ANOVA; Tukey’s test for multiple comparisons.
(TIF)

S1 Table Complementary DNA PCR primer sequences. All primer sequences used in the study are listed.
(TIF)
