# Supplementary figures and images for: A transient DMSO treatment increases the differentiation potential of human pluripotent stem cells through the Rb family
Source: PLoS One. 2018 Dec 12;13(12):e0208110. doi: 10.1371/journal.pone.0208110 (PMC6291069; doi:10.1371/journal.pone.0208110)

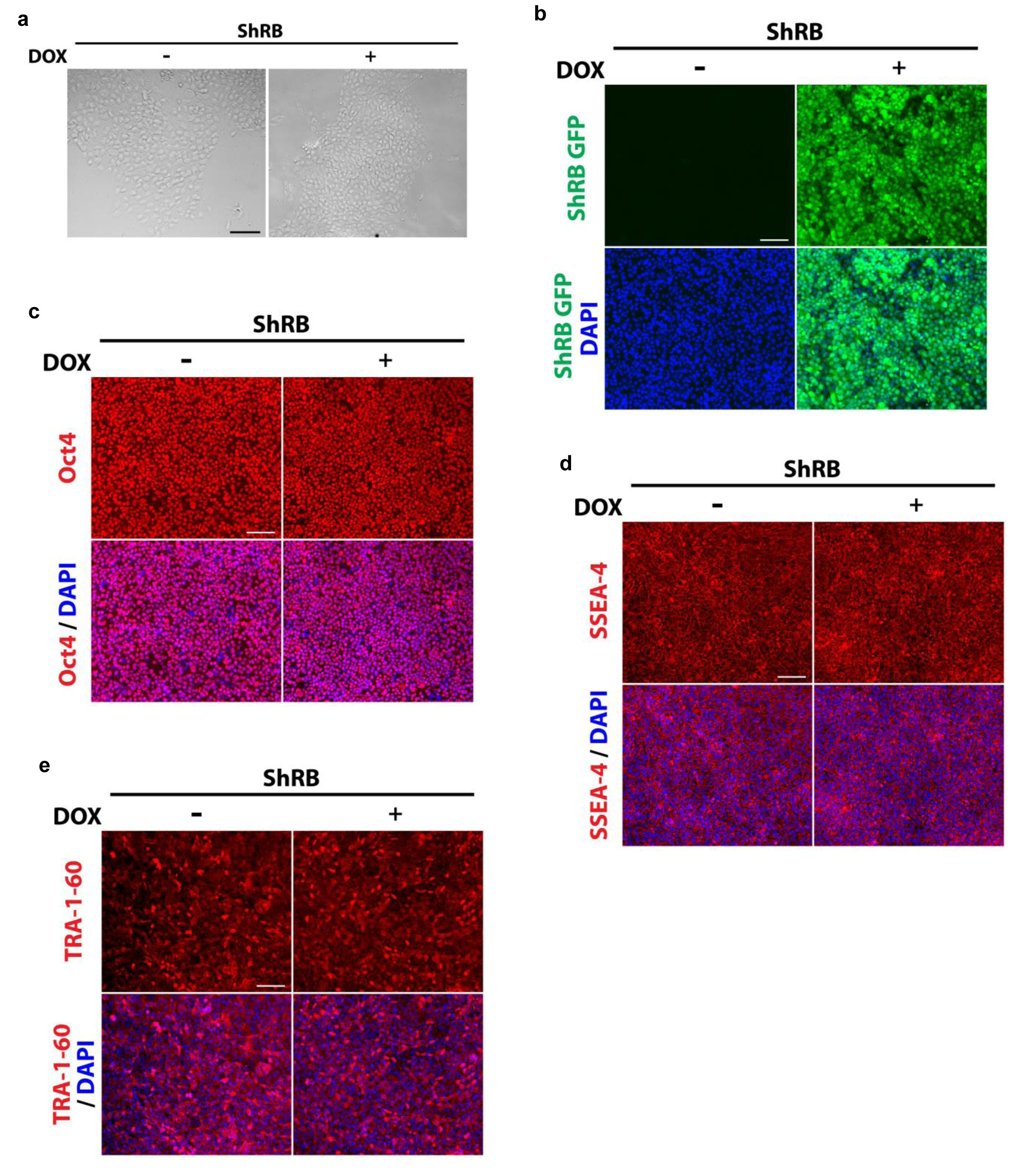

Supplement: S1 Fig — Representative images of (a) phase-contrast hPSC colonies and (b) GFP expression following DOX induction of the ShRB cell line. Immunostaining for (c) Oct4, (d) SSEA-4, and (e) TRA-1-60 in control (-DOX) and ShRB (+DOX) cells. Scale bars, 100 μm. (TIF) [file pone.0208110.s001.tif]

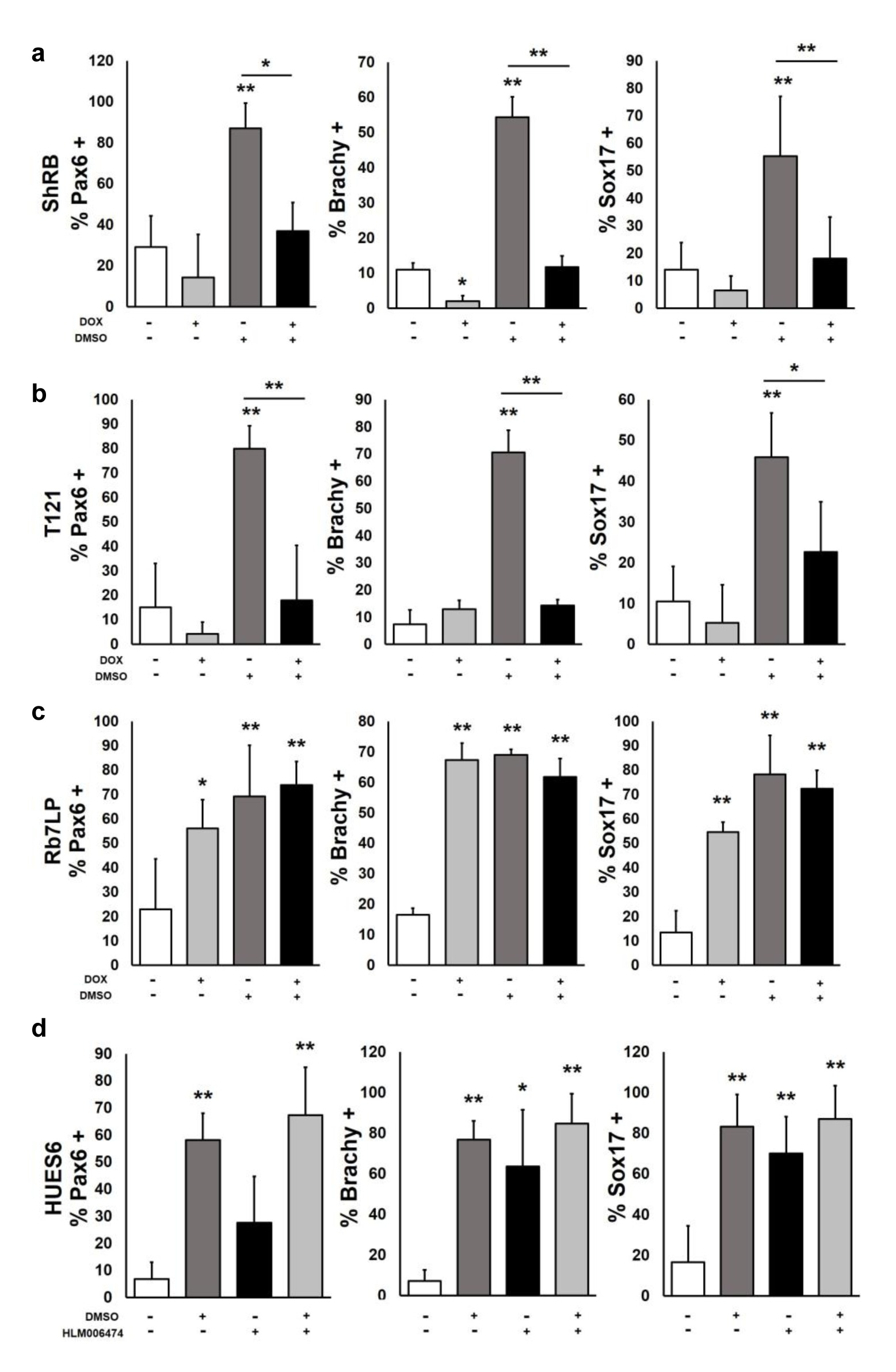

Supplement: S2 Fig — Percentage of hPSCs differentiating into Pax6+ ectodermal, Brachyury (Brachy)+ mesodermal, or Sox17+ endodermal cells following directed differentiation into each germ layer in the (a) ShRB, (b) T121, and (c) Rb7LP cell lines with and without DOX treatment and a 24h 2% DMSO treatment and the (d) HUES6 cell line pre-treated with and without 30μM E2F inhibitor HLM006474 and a 24h 2% DMSO treatment. Error bars, s.d. of 3–6 biological replicates. * p ≤ 0.05, ** p ≤ 0.01 under one-way ANOVA; Tukey’s test for multiple comparisons. (TIF) [file pone.0208110.s002.tif]

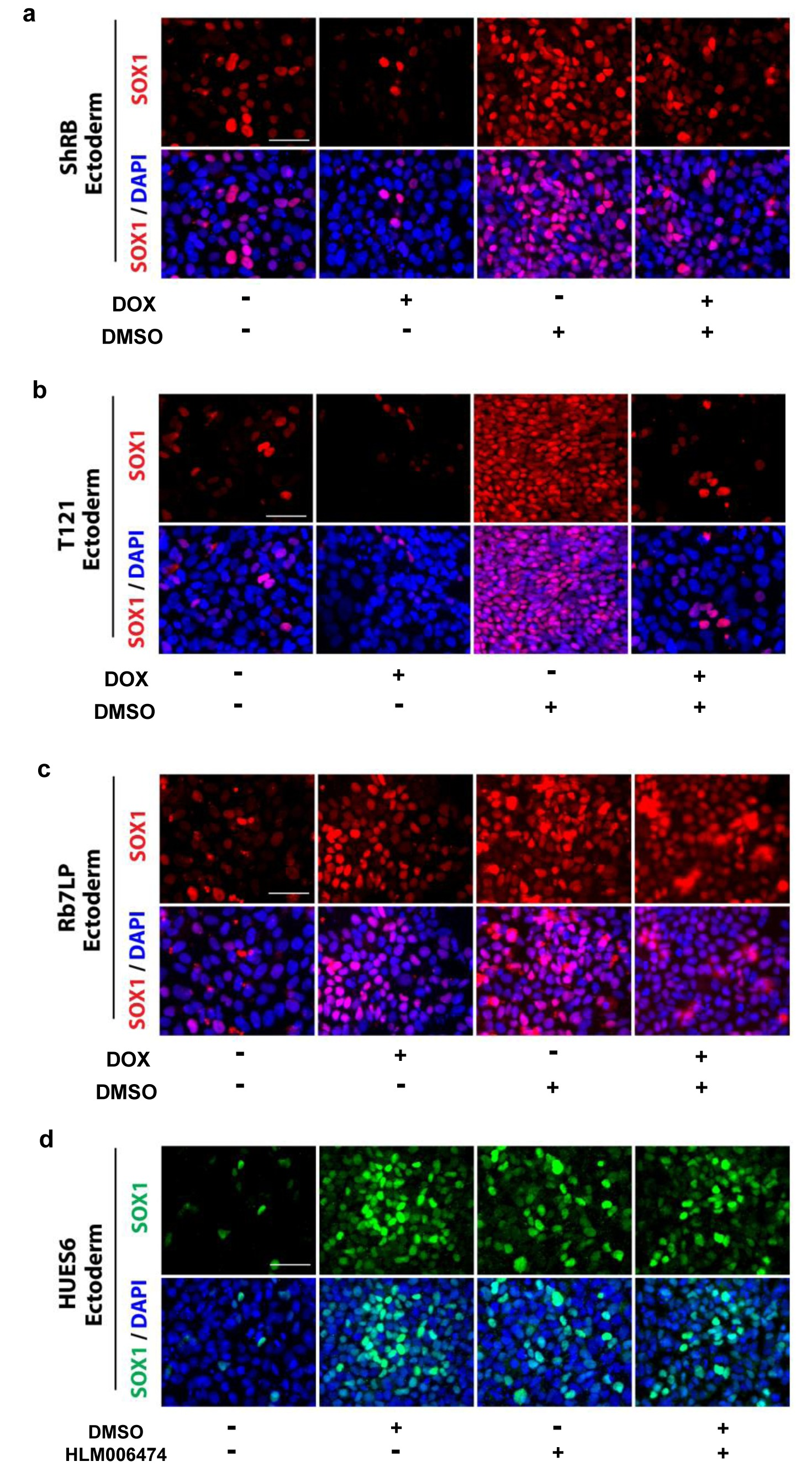

Supplement: S3 Fig — Immunostaining for Sox1 following directed differentiation of the (a) ShRb, (b) T121, and (c) Rb7LP cell lines into the ectodermal germ layer. (d) Immunostaining for Sox1 following directed differentiation into the ectodermal germ layer of the HUES6 cell line pre-treated with and without 30μM E2F inhibitor HLM006474 and a 24h 2% DMSO treatment. (TIF) [file pone.0208110.s003.tif]

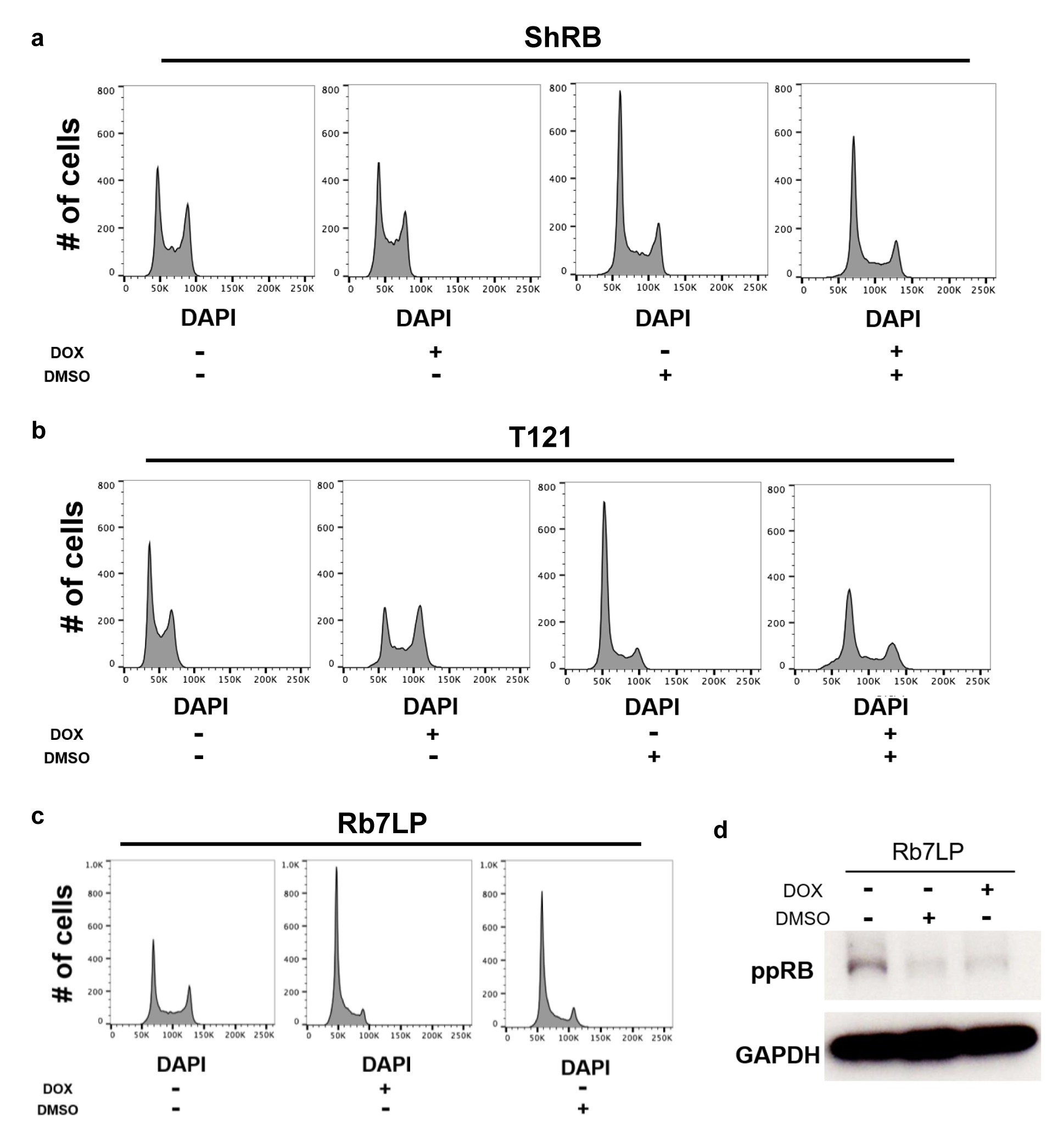

Supplement: S4 Fig — Distribution of hPSCs in the G1, S, and G2/M phases of the cell cycle in the (a) ShRB, (b) T121, and (c) Rb7LP cell lines with and without DOX treatment and a 24h 2% DMSO treatment. (d) Western blot showing the levels of hyperphosphorylated Rb in Rb7LP cells with and without DOX treatment compared to DMSO-treated cells. ppRB, hyperphosphorylated Rb. GAPDH serves as a loading control. (TIF) [file pone.0208110.s004.tif]

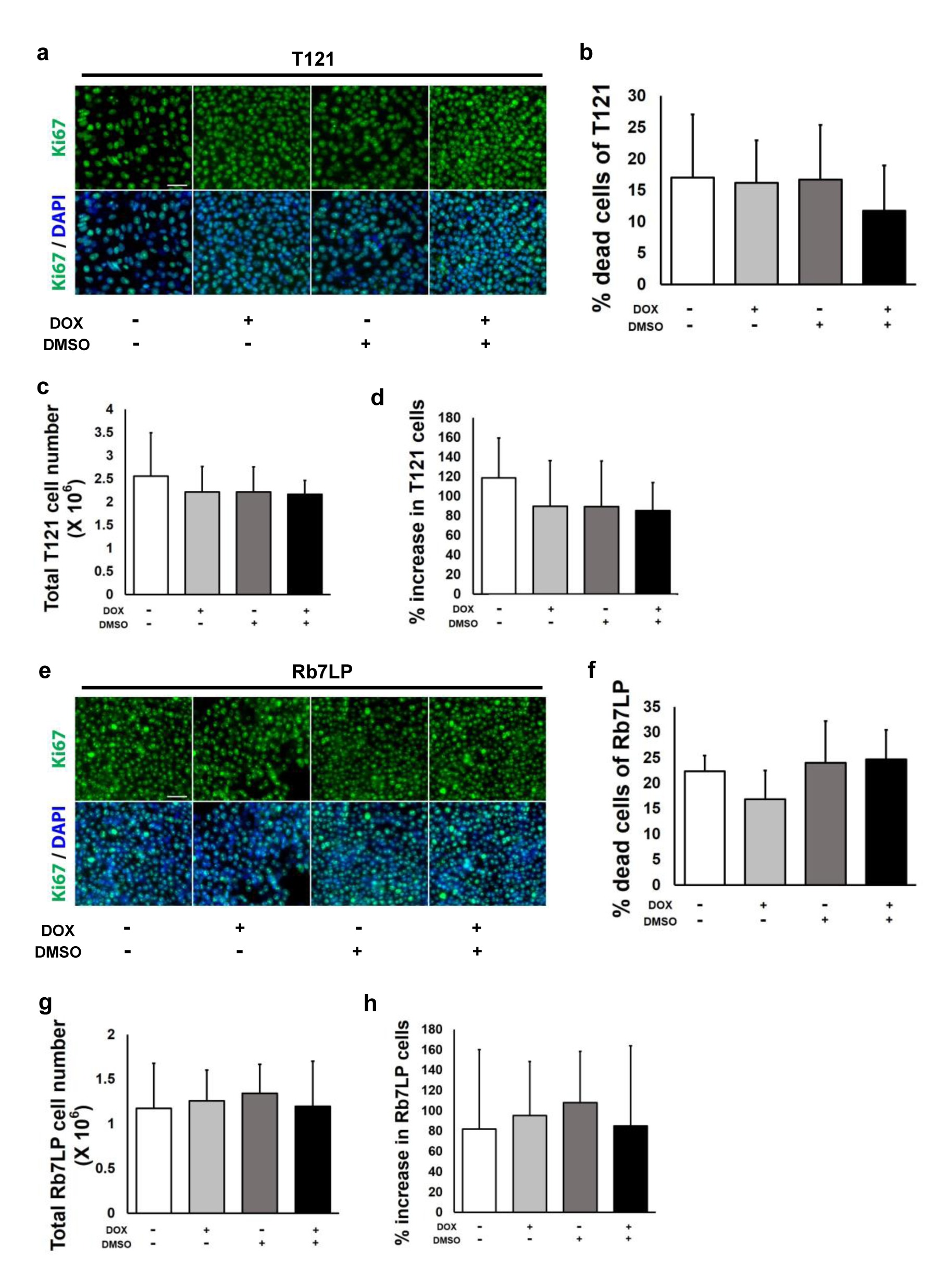

Supplement: S5 Fig — (a) Immunostaining for the proliferation marker Ki67 in T121 cells with and without DOX treatment and a 24h 2% DMSO treatment. (b) Percentage of dead cells of T121 cells following treatment with and without DOX and a 24h 2% DMSO treatment using the trypan blue exclusion assay. (c) Total cell numbers of T121 cells following treatment with and without DOX and a 24h 2% DMSO treatment. (d) Percentage increase in total cell number following treatment with and without DOX and a 24h 2% DMSO treatment relative to initial plating density in the T121 cell line. (e) Immunostaining for Ki67 in Rb7LP cells with and without DOX treatment and a 24h 2% DMSO treatment. (f) Percentage of dead cells of Rb7LP cells following treatment with and without DOX and a 24h 2% DMSO treatment using the trypan blue exclusion assay. (g) Total cell numbers of Rb7LP cells following treatment with and without DOX and a 24h 2% DMSO treatment. (h) Percentage increase in total cell number following treatment with and without DOX and a 24h 2% DMSO treatment relative to initial plating density in the Rb7LP cell line. Error bars, s.d. of 3–6 biological replicates. (TIF) [file pone.0208110.s005.tif]

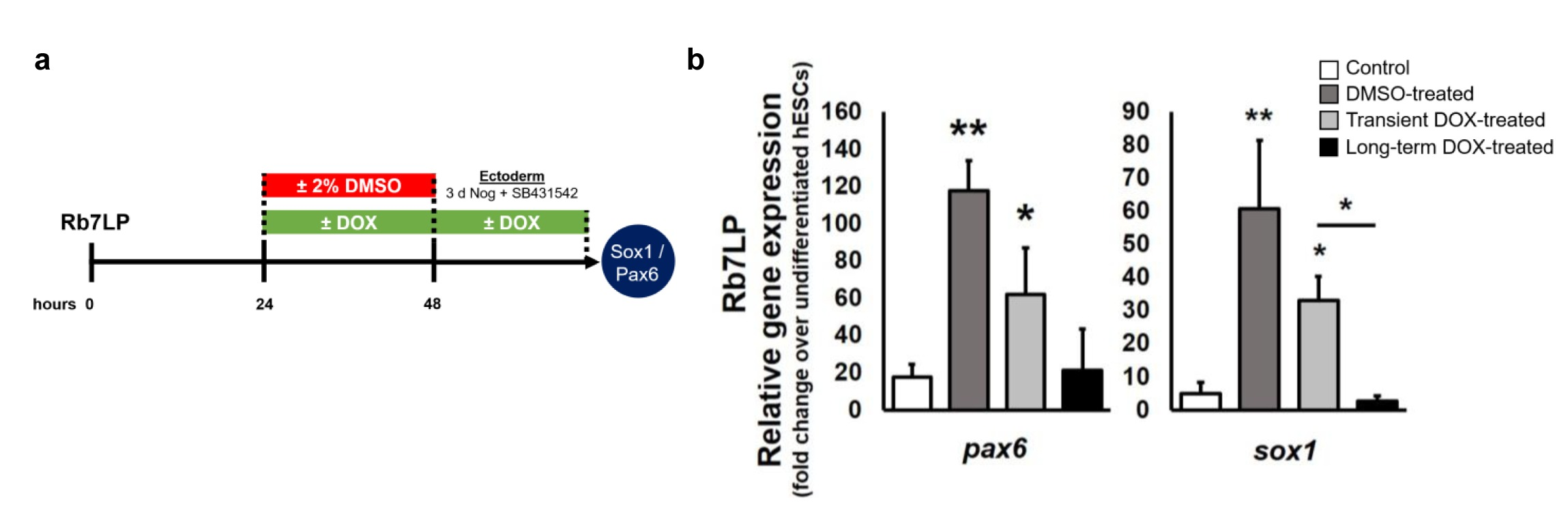

Supplement: S6 Fig — (a) Directed differentiation into the ectodermal germ layer of the dox-inducible Rb7LP cell line, which expresses the active non-phosphorylatable form of Rb, and compared with control and 2% DMSO-treated cells. Treatment with DOX was for 24h prior to directed differentiation (Transient DOX-treated) or for 24h prior to directed differentiation and throughout the ectodermal differentiation (Long-term DOX-treated). (b) Quantitative RT-PCR analyses of sox1 and pax6 expression following differentiation into the ectodermal germ layer. Error bars, s.d. of 3–5 biological replicates. * p ≤ 0.05, ** p ≤ 0.01 under one-way ANOVA; Tukey’s test for multiple comparisons. (TIF) [file pone.0208110.s006.tif]

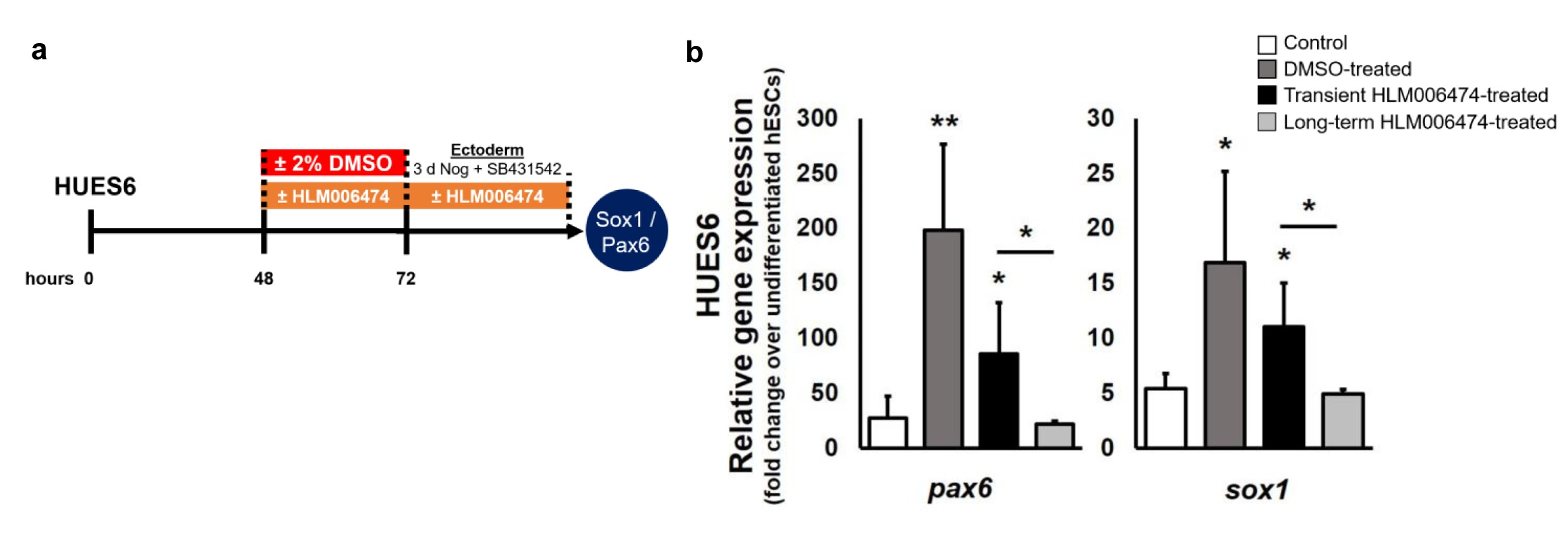

Supplement: S7 Fig — (a) Directed differentiation into the ectodermal germ layer of HUES6 cells treated with HLM006474 compared with control and 2% DMSO-treated cells. Treatment with HLM006474 was for 24h prior to directed differentiation (Transient HLM006474-treated) or for 24h prior to directed differentiation and throughout the ectodermal differentiation (Long-term HLM006474-treated). (b) Quantitative RT-PCR analyses of sox1 and pax6 expression following differentiation into the ectodermal germ layer. Error bars, s.d. of 2–5 biological replicates. * p ≤ 0.05, ** p ≤ 0.01 under one-way ANOVA; Tukey’s test for multiple comparisons. (TIF) [file pone.0208110.s007.tif]

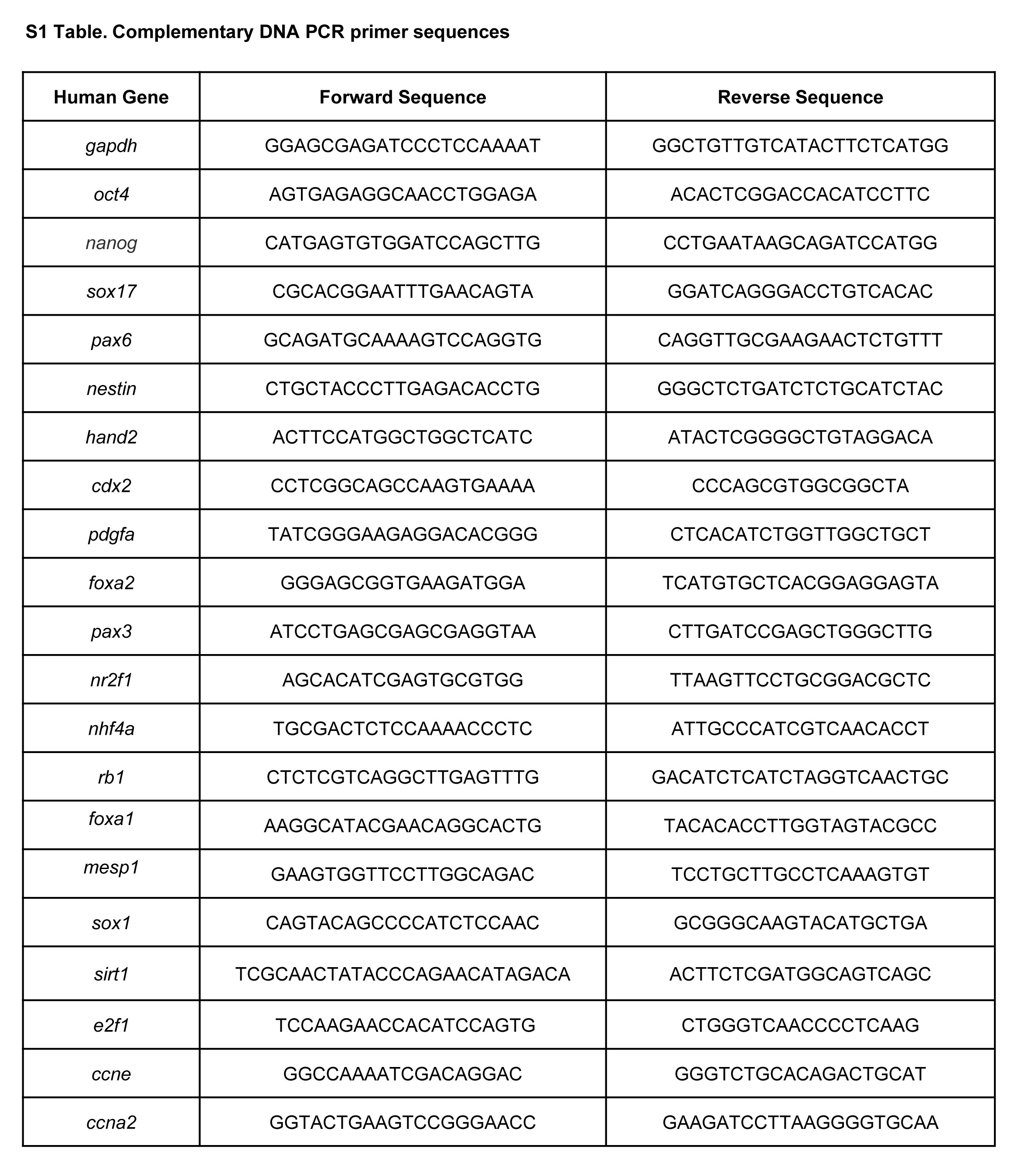

Supplement: S1 Table — All primer sequences used in the study are listed. (TIF) [file pone.0208110.s008.tif]
